# Healthcare providers' perspectives on collaboration of care for acute cystitis in women and the role of the community pharmacy: A qualitative study

**DOI:** 10.1016/j.rcsop.2025.100694

**Published:** 2025-12-06

**Authors:** Marle Gemmeke, Maureen C. Jansen, Thomas G.H. Kempen, Marcel L. Bouvy

**Affiliations:** a Division of Pharmacoepidemiology and Clinical Pharmacology, Utrecht Institute for Pharmaceutical Sciences, Faculty of Science, Utrecht University, Utrecht, the Netherlands; b Department of Pharmacy, Uppsala University, Uppsala, Sweden

**Keywords:** Urinary tract infection, Primary care, Prescribing pharmacist, Collaboration, Task shifting

## Abstract

**Introduction:**

Acute cystitis (AC) is generally treated in primary care by general practitioners. Support from the community pharmacy may relieve the GP's workload and increase accessibility of care for women.

**Aim:**

To explore healthcare providers' (HCPs') perspectives on the collaboration of care for AC and investigate their views on possibilities for task shifting towards community pharmacy.

**Method:**

This qualitative study was conducted within the setting of pharmacotherapy audit meetings (PTAMs) involving general practitioners and community pharmacists. Focus group discussions were embedded in PTAMs to gain insight into participants' perspectives on the organization of care for AC and the potential role of the community pharmacies. Additionally, individual interviews were carried out with a subset of PTAM participants and other primary care professionals (e.g. assistants). Focus groups and interviews were audio-taped, transcribed verbatim and thematically analyzed.

**Results:**

Five focus groups and 12 interviews were conducted; in total 57 participants were included (45 of them were working in general practice). Although HCPs were experienced in providing AC care, they reported some shortcomings in the current care process. They perceived limited advantages in shifting tasks to community pharmacies. Key barriers included time constraints within pharmacies and insufficient access to patients' medical histories. Expanding the role of community pharmacies could be supported through financial compensation for counseling, adequate training of pharmacy personnel, and strong collaboration between pharmacists and general practitioners.

**Conclusion:**

Although there is room for improvement in the organization of care for AC, participants saw limited opportunities for task shifting towards community pharmacy.

## Highlights

• Risk of false-negative diagnoses and quality of advice about preventive and selfcare measures are areas of improvement.
• Major barriers for task shifting include a limited access to patient’s files, lack of privacy, and costs for the pharmacy.
• Community pharmacies can have an important role in signaling of long-term antibiotic prophylactic treatment.

## 1 Introduction

An uncomplicated urinary tract infection (UTI), also called acute cystitis (AC), is one of the most prevalent reason for a woman to contact a general practitioner (GP).^1^ More than 50 % of the women experience at least one UTI during their life.^2^ In 20–40 % of these cases, AC presents itself in a recurrent manner, meaning at least three episodes occur within 12 months.^3^ AC often impacts women's daily activities. For example, it leads to reduced capacity for work and reduced social activities, therefore it affects quality of life, especially in women who experience recurrent AC.^4^, ^5^, ^6^ This underlies the need for fast diagnosis and accurate treatment.

In most Western countries, GPs experience a high workload, leading to a lack of time for their patients.^7^ Task shifting to other healthcare providers (HCPs), e.g. community pharmacists (CPs), may relieve the GP's workload. The shift involves redistribution of tasks to HCPs with other qualifications, which usually are not within their scope of work.^8^

In some countries, like Canada, New Zealand and the United Kingdom, CPs have the authority to diagnose AC and prescribe antibiotics if necessary.^9^ Evidence suggests that pharmacist-led AC management is safe and effective.^10^ In the Netherlands, pharmacy staff is trained to advise patients about self-management and treatment with certain minor ailments, e.g. diarrhea, headache and hemorrhoids.^11^ Therefore, diagnosis and treatment of other minor ailments, e.g. AC, is possibly a field that is suitable for task shifting. Yet, in the Netherlands, the GP is currently responsible for the diagnosis and treatment initiation of AC.^12^ Previous research shows that almost 70 % of Dutch GPs find that CPs could support women with AC with recommendations for prevention.^13^ Half of these GPs indicated that CPs could help determine whether someone is eligible for prophylactic treatment.

Certain requirements for task shifting of AC care to the community pharmacy have been identified in previous research. According to GPs and CPs, these requirements include development of a referral pathway, discussing and possibly adapting to GPs' concerns, making agreements, a pharmaceutical protocol for managing and treating AC, a questionnaire to verify patient symptoms, and a training program.^13^^,^^14^

The diagnosis of AC is typically based on clinical symptoms, supported by point-of-care tests for leucocytes and nitrite in (morning) urine.^15^ Although the test can be performed quickly, women often need to wait several hours for the results and interpretation by the GP. The combined urine test on leucocytes and nitrites has a high sensitivity and specificity for detecting an infection, however its performance on ruling out an infection is lower.^16^^,^^17^ This results in a relatively high number of false negative results. In case of clinical symptoms with a negative nitrite test, sometimes additional tests are performed, delaying the AC diagnosis for another day. Recent research shows that a diagnosis with the Acute Cystitis Symptom Score (ACSS) questionnaire is as reliable as urine testing and may result in less delays of treatment.^18^

To date, limited research has been conducted on whether the community pharmacy can take over some roles regarding AC care to relieve the GP's workload.^13^^,^^14^ Additional research is needed to explore how task shifting in the collaboration of AC care towards the community pharmacy can be implemented.

### 1.1 Aim of the study

The aim of the study was to investigate HCPs' perspectives on the collaboration of care for AC and investigate their views on possibilities for task shifting towards community pharmacy.

## 2 Methods

### 2.1 Study design

**Fig. 1:**
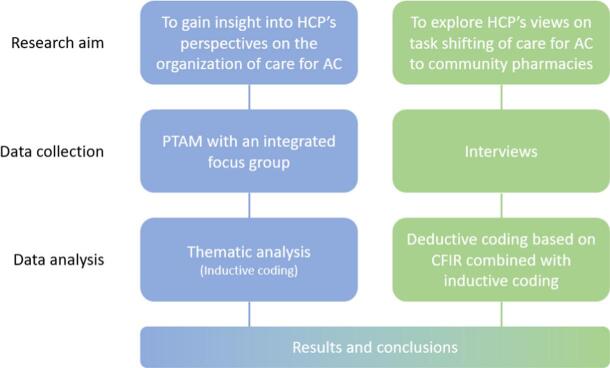
Overview of research methods and qualitative data analysis. Abbreviations: AC, Acute cystitis; PTAM, Pharmacotherapeutic audit meeting; CFIR, Consolidated Framework for Implementation Research.

We embedded focus group discussions within the setting of pharmacotherapy audit meetings (PTAMs) involving general practitioners and community pharmacists (Fig. 1). Additionally, individual interviews were carried out with a subset of PTAM participants and other primary care professionals. In the Netherlands, PTAMs are an opportunity for GPs and CPs to exchange information and views about pharmacotherapy. Additionally, new treatment policies may be discussed, and agreements can be made. The aim of these meetings is to improve the prescribing and dispensing of drugs. In the PTAM related to this study, a training to inform participants about the current guidelines for diagnosis and treatment of AC was combined with a focus group discussion.

The Consolidated criteria for Reporting Qualitative research (COREQ) checklist was used to report the study *(Appendix A).*^19^ Data were collected from October 7th, 2024, until December 9th, 2024.

### 2.2 Participants

CPs and GPs who collaborate in so-called PTAM groups were approached through the researchers' network and were invited to join the study via e-mail. From these groups, individual GPs and CPs were invited for the interviews. All participants received an invitation letter and were free to invite their assistants to participate in the PTAMs and/or interviews as well. All participants gave informed consent. From each PTAM-group, at least one GP and one CP was asked to participate in the interview. Specifically, we also aimed to include GP assistants in the interviews.

### 2.3 Data collection

Demographic data of the participants were collected (e.g. gender, position, and years of experience).

#### 2.3.1 Pharmacotherapy audit meetings with focus groups

A training module on AC was developed to inform participants about the Dutch GP guideline and the organization of AC care. Its format was based on the PTAM user guide and module from the Dutch Institute for Rational Use of Medicine (IVM), the Dutch GP guideline (NHG) and additional references. [12, 20] Based on previous research we developed a conversation guide (*Appendix B)* for the focus group sessions embedded in the PTAM session.^13^^,^^14^^,^^18^^,^^21^^,^^22^ The following themes were discussed: time used for diagnosis and treatment, experiences with diagnosing, decisions on whether or not to start antibiotic treatment, non-pharmacotherapeutic recommendations, prophylactic treatment, role of the community pharmacy, agreements. One researcher (MJ) presented the module and thus informed participants about the guideline, whereas another researcher (MG or MB) led the focus group discussions. The PTAMs were scheduled for 1 to 1.5 h.

#### 2.3.2 Individual interviews

The Consolidated Framework for Implementation Research (CFIR^23^; was used to develop a semi-structured interview guide *(Appendix C)*. This framework assesses all domains important for implementation of a new care process or intervention in practice. Thus, it was used to gather, recognize, and thematize barriers and facilitators for implementation of such an innovative care process. All five domains of CFIR were used in the interview guide and can be found in Table 1.

**Table 1:** Shifting AC care towards the community pharmacy: CFIR domains coupled to themes analyzed.

CFIR domain	Theme
Innovation	Diagnosis and treatment initiation in the community pharmacy
Implementation process	Requirements for implementation
Inner setting	The general practice and the community pharmacy
Individuals	CPs and their technicians
Outer setting	Interprofessional collaboration Patients with recurrent AC

Abbreviations: ACSS, Acute Cystitis Symptom Score; GP, General practitioner; CP, Community pharmacist; AC, Acute cystitis.

For the interviews, a schematic design of an innovative care process for AC was presented to the participants *(Appendix D)* and included the following: Patients with recurring AC 1) fill out the ACSS questionnaire in the community pharmacy for diagnosis, 2) are diagnosed by a CP or their technician, 3) receive selfcare/lifestyle/preventive recommendations, 4) are prescribed antibiotics and dispensed if needed, 5) the GP is informed about diagnosis and treatment and approves the prescription. The maximum duration of the interviews was 20 min.

### 2.4 Data analysis

The PTAMs and interviews were recorded with a voice-recorder and transcribed verbatim afterwards. The PTAMs and interviews were analyzed separately with the use of NVivo 15. Demographic data was analyzed descriptively.

#### 2.4.1 Pharmacotherapy audit meetings with focus groups

Thematic analysis was used to analyze the PTAM transcripts. Inductive data coding was conducted independently by two researchers (MJ, MG). The researchers discussed the codes and divided them into categories and umbrella themes, which were processed into a coding tree. Differences in codes between the two researchers were discussed and if needed presented to a third researcher (TK).

Multiple themes were identified from the focus group sessions during the PTAMs. Themes that were relevant for answering the research question were included in the analysis, irrelevant themes were excluded. Some themes from the focus group sessions had significant overlap with those in the individual interviews. Those were only included in the interview analysis, as the interviews provided more in-depth information.

#### 2.4.2 Individual interviews

A topic list based on the domains of CFIR was developed before analysis, in order to guide the coding (deductive coding). Table 1 provides an overview of these topics based on CFIR. Inductive coding was also used, since new topics emerged during the interviews. Coding was performed independently by two researchers (MJ, MG).

### 2.5 Ethical considerations

The study protocol was approved by the Institutional Review Board of the Division of Pharmacoepidemiology and Clinical Pharmacology, Utrecht University (reference number: UPF2409, date of approval 14 May 2024). All participants gave written or oral consent before participating in the PTAMs and interviews. All identifiable data was removed from the transcripts. Transcripts were stored under a research number and identifiable data was stored separately.

## 3 Results

Five PTAMs were held. In total, this resulted in 54 participants (range: 7 to 21 per PTAM) in the focus group sessions. Nine of them also participated in the interviews. Additionally, three interview participants were included who did not attend the PTAM but were working in one of the general practices or pharmacies of the PTAM-groups. Thus, a total of twelve participants were interviewed. The demographic characteristics of the participants are summarized in Table 2. Most were female (64.8 % and 75.0 % in the PTAMs and interviews, respectively) and most of the participants were working in general practice (77.8 % and 66.7 % in the PTAMs and interviews, respectively).

**Table 2:** Demographic characteristics of participants.

	PTAMs (*n* = 54)	Interviews (*n* = 12)
Female gender *(N, %)*⁎	35 *(64.8 %)*	9 *(75.0 %)*
Male gender *(N, %)*⁎	18 *(33.3 %)*	3 *(25.0 %)*
Years of work experience *(median [Q1 – Q3])*	11 *[4–18]*	10 *[4–17]*
Position *(N, %)*		
General practitioner	36 *(66.7 %)*	5 *(41.7 %)*
Other in general practice ⁎⁎	6 *(11.1 %)*	3 *(25.0 %)*
Community pharmacist	11 *(20.4 %)*	4 *(33.3 %)*
Other in community pharmacy ⁎⁎⁎	1 *(1.9 %)*	

From the PTAM-groups, 9 participants also participated in the interviews.

⁎ One participant did not fill in their gender.

⁎⁎ GP assistants (in training), physician assistant, nurse, practice-based pharmacist.

⁎⁎⁎ Pharmacist in training. Abbreviations: PTAM, Pharmacotherapeutic audit meeting; GP, General practitioner.

### 3.1 Pharmacotherapy audit meetings with focus groups

During the PTAMs the HCPs' perspectives on and their experiences with the collaboration of AC care were explored. Table 3 shows an overview of the themes analyzed. The full codebook can be found in *Appendix E*.

**Table 3:** Organization of care for AC: Themes emerged from the focus group discussions.

*Theme 1:* Diagnosis	*Theme 3:* Recurrent acute cystitis
Duration of symptoms before diagnosis	Attention for selfcare and preventive recommendations
Time to diagnosis (same day or later)	Painkillers and self-care products
Reliability of urine test	Considerations to initiate prophylaxis
Risks of symptom-based diagnosis	Evaluation of prophylaxis

***Theme 2:* Choice of treatment**	***Theme 4:* Role of the community pharmacy**

Wait-and-see policy	Dispensing antibiotics
Postponed prescription	Provision of selfcare and preventive recommendations
Antibiotic treatment	Signaling function
Considerations when deciding on treatment	Opportunities for task shifting

Abbreviations: GP, General practitioner; AC, Acute cystitis.

### 3.2 Diagnosis

In some practices, general practice assistants ask their patients how long they have experienced symptoms when they bring their urine. However, most GPs mentioned that it was unclear to them for how long the AC symptoms had persisted when patients reached out to them. Most assumed that patients with recurrent AC tend to contact the GP faster than patients with a first episode. All GPs preferred to diagnose AC by means of urine testing, compared to symptom-based diagnosis.

**Participant 4 (GP):***“The test is quite simple, so why would you not use it? The results come out quickly.”*

The urine test usually provides same-day results. Urine quality affects reliability of results, thus delays occur when no morning urine is provided or when patients urinated multiple times during the night, resulting in unknown bladder retention time. GPs reported frequent negative nitrite tests, often requiring follow-up testing.

Some GPs relied on symptom-based diagnosis, especially when symptoms where very clear or when logistical barriers prevented urine submission. Both GPs and CPs noted that this approach risks unnecessary antibiotic prescribing, though one GP downplayed this concern.

**Participant 22 (GP):***“There is definitely one prescription a year that is not right, but I can live with that.”*

#### 3.2.1 Choice of treatment

GPs said that a positive urine test, and thus an AC diagnosis, led to antibiotic treatment in most cases. Opinions about wait-and-see policies differed among GPs.

**Participant 10 (GP):***“I think wait-and-see policies are most commonly seen when women decide to wait themselves, which is off our radar. If women take the effort to hand in their urine, most of the time they are really suffering, thus prefer to do something about it.”*

A minority of GPs offered postponed prescription, advising patients to delay filling of the antibiotic prescription and try self-care first. However, many GPs doubted patients would wait and CPs believed most prescriptions were ultimately filled.

#### 3.2.2 Recurrent acute cystitis

GPs reported providing self-care and preventive advice to patients with recurrent AC, sometimes supported with leaflets or website referrals. They believed assistants routinely advised hydration, with mixed assumptions about recommending painkillers. Most GPs and CPs did not recommend over-the-counter (OTC) products like cranberry and D-mannose but they were aware some patients are use these.

Most GPs prescribed prophylaxis in line with the Dutch GP UTI guidelines. Some evaluated and stopped prophylaxis after a few months, while others applied less strict follow-up, leading to prolonged antibiotic use. Some GPs assumed that some patients are occasionally not evaluated. One CP monitored long-term prophylaxis.

**Participant 13 (CP):***“If I see antibiotic prophylactic treatment is used on the long term, I will send an email about it to the GP, or I use Microsoft Teams nowadays.”*

#### 3.2.3 Role of community pharmacy

Both GPs and CPs agreed that community pharmacies currently contribute to AC care mainly through antibiotic dispensing. This includes providing instructions on use, possible side effects, and occasionally advising increased fluid intake. Opinions differed on the potential role of pharmacies in self-care and prevention. Some GPs and CPs considered repeating recommendations unnecessary, while others viewed it as beneficial.

Multiple GPs saw an important role for CPs in identifying patients whose long-term antibiotic prophylactic treatment should be evaluated.

**Participant 10 (GP):***“It is really helpful to us if the pharmacist is signaling: ‘You are prescribing prophylaxis for the third time in a row’.”*

Almost all HCPs doubted if the community pharmacy should have a role in diagnosing and treatment initiation of AC. Most believed this should remain part of the GP's job.

**Participant 49 (GP):***“It is not part of the care task of a pharmacist.”*

**Participant 20 (CP):***“It would be difficult for me to diagnose AC if I don't have access to the medical history. Particularly, this applies to recurrent cystitis and if I have no clue about previous symptoms.”*

### 3.3 Individual interviews

**Fig. 2:**
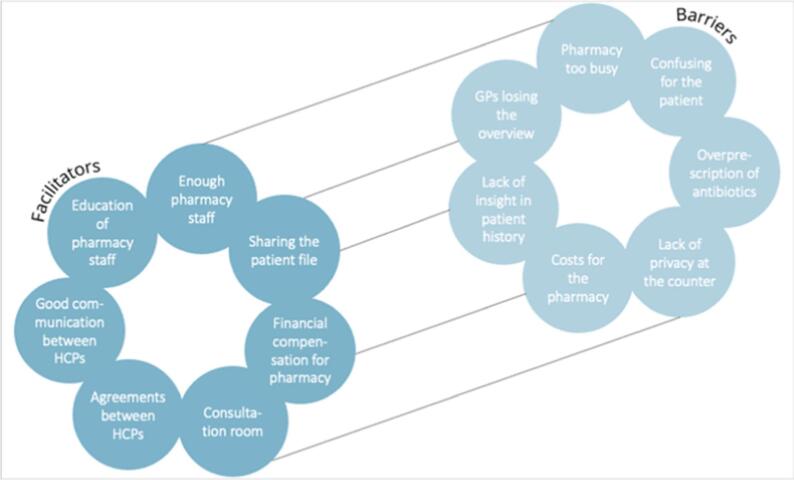
Barriers and facilitators for task shifting AC care towards the community pharmacy, identified from the individual interviews across all CFIR domains. Abbreviations: CFIR, Consolidated Framework for Implementation Research; HCP, Healthcare provider; GP, General practitioner

During the interviews, individual perspectives on task shifting in the care process for AC were assessed. The themes analyzed can be found in Table 1. The barriers and facilitators for task shifting towards the community pharmacy are shown in Fig. 2.

#### 3.3.1 Diagnosis and treatment initiation in the community pharmacy (CFIR domain: innovation)

Most GPs and CPs identified reduced GP workload as the main advantage of shifting diagnosis and treatment initiation to community pharmacies. Some also noted benefits for pharmacy staff, particularly that new responsibilities could make their work more engaging.

**Participant 17 (CP):***“I think it is nice, an expansion of tasks for the community pharmacy.”*

Both groups saw potential value in using the ACSS questionnaire for diagnosis, citing greater patient convenience and possible cost savings compared to urine testing.

**Participant 41 (CP):***“From the point of view of the patient, it will be much easier for them if both diagnosis and treatment initiation can take place at the pharmacy. For patients, the pharmacy's opening hours are usually also more convenient than the general practice's opening hours.*

However, concerns were also raised. For example, a negative ACSS result still requires urine submission.

**Participant 26 (GP):***“This is an extra step for those patients.”*

Additional barriers included risk of misdiagnosis, potentially leading to excessive antibiotic prescribing. Also, loss of GP oversight of patient files, potential questionnaire manipulation, and added costs for pharmacies were mentioned. Some assistants also noted the time burden as a barrier to the pharmacy. Overall, most GPs and CPs did not perceive clear advantages of shifting AC care to community pharmacies, including ACSS use.

**Participant 7 (GP in training):***“I have doubts if this would improve healthcare. So, you must prove this first.”*

**Participant 41 (CP):***“I think there will be resistance from GPs. GPs see the urine test as the golden standard to diagnose AC. Also, even though I am not worried about it myself, I think GPs are worried that task shifting will lead to an increased number of antibiotic prescriptions, due to enhanced access to antibiotic treatment.”*

#### 3.3.2 Requirements for implementation (CFIR domain: implementation process)

Making agreements was seen as a key facilitator for implementing AC services in community pharmacies. Some GPs and CPs suggested using PTAMs to establish or evaluate such agreements. Sharing patient files was considered essential, along with financial compensation and sufficient staffing.

**Participant 17 (CP):***“If the staff is adequate, it should be possible.”*

Several GPs emphasized the need for clear protocols for diagnosis, treatment, and referral. Finally, patients should be informed that they can also consult the community pharmacy instead of the GP.

#### 3.3.3 The general practice and the community pharmacy (CFIR domain: inner setting)

Some GPs said that providing AC care takes quite some time from their assistants. However, they supposed that their assistants enjoy urine testing and want to keep this task.

**Participant 24 (GP):***“I think that this is time-consuming for our assistants, but also a nice variety to their work. So, they can do something practical instead of being on the phone all day.”*

Some GPs and CPs questioned whether community pharmacies are the most suitable setting for AC diagnosis and treatment. A shortage of pharmacy technicians was frequently mentioned as a barrier.

**Participant 20 (CP):***“Currently, we are dealing with staff shortages. In such circumstances, expansion of tasks is not prioritized.”*

In addition, some GPs noted that patients may feel uncomfortable receiving recommendations at the counter, highlighting the need for a consultation room.

#### 3.3.4 CPs and their technicians (CFIR domain: individuals)

Most GPs and CPs believed the pharmacy technician could have an essential role in providing such an AC service. They were seen as capable of providing lifestyle advice, supporting patients in completing the ACSS questionnaire, and interpreting results if properly trained. Some HCPs stressed the need for CP supervision.

**Participant 52 (CP):***“I think that the pharmacy technician can do this, but there should be supervision.”*

One CP argued the pharmacist, not the technician, should hold this role. Concerns were also raised about technicians' current clinical knowledge, highlighting the need for additional education on UTIs and AC.

#### 3.3.5 Patients with recurrent AC (CFIR domain: outer setting)

Limited health literacy was identified as a barrier to using the ACSS questionnaire. One GP doubted patients' overall ability to self-assess accurately.

**Participant 26 (GP):***“I think it is a very bad idea. We have a lot of people that think they have a UTI, but they do not.”*

HCPs also mentioned that since patients are used to visiting the GP if they experience AC symptoms, it will take some time before patients are aware they can also visit the community pharmacy instead.

**Participant 26 (GP):***“I do not think it is routine for the patient to ask something in the community pharmacy when they think they have a UTI.”*

#### 3.3.6 Interprofessional collaboration (CFIR domain: outer setting)

Collaboration between GPs and CPs was generally viewed positively, with good communication seen as key to successful implementation. Additional IT support and financial compensation for pharmacies were mentioned as requirements. However, many CPs believed GPs would be reluctant to shift tasks to pharmacies.

**Participant 17 (CP):***“I have doubts if GPs are willing tosharethe patients' medical historywithus, which I believe is needed to be able to diagnose safely and accurately.”*

## 4 Discussion

This study shows that while GPs and their assistants are experienced in providing AC care, there is still room for organizational improvements. GPs noted risks of a false-negative urine tests and were uncertain about the consistency of advice given by their assistants. Despite these issues, both GPs and CPs were reluctant to shift AC care to community pharmacies, citing barriers such as limited access to the patient's medical files, lack of privacy in the pharmacy, and lack of financial compensation.

These findings are in line with previous research in Singapore about GPs' perceptions of the CP's role and GPs' willingness to collaborate with CPs in primary care.^24^ Although GPs in that study appreciated their general collaboration with CPs, they were doubtful about clinical collaborations specifically. GPs' major concerns about such clinical collaborations were: the absence of a shared patient electronic health record system, extra time and costs of pharmacy-led services, and conflicts with GPs' own business interests. With the exception of the business interest, similar concerns came forward in our study.

Previous research on collaboration between GPs and CPs highlighted the need for consultation rooms, role clarification education, pharmacy protocols, and agreements between GPs and CPs to enable integrated AC care.^13^^,^^25^ A previous systematic review further stressed standardized pharmacy staff training and access to shared patient information as prerequisites.^10^ Our findings confirmed and additionally identified funding and staffing needs, consistent with an English study on pharmacy-led UTI service, which also noted reduced GP workload as a possible advantage of task shifting.^14^ Consistent with earlier Dutch research, GPs in our study reported routinely prescribing antibiotics for AC, rarely using postponed prescriptions. Most GPs believed patients preferred immediate antibiotic and doubted compliance with delayed treatment advice.^26^

Our research indicates that GPs and their assistants are currently falling short of providing patients with a complete overview of relevant selfcare and preventive recommendations for AC. Dutch research on women's familiarity with those recommendations showed that the majority of participants had limited awareness of selfcare and prevention.^22^ A previous survey study among Dutch GPs and CPs indicated that almost 100 % of the CPs believed they were capable to provide their patients information on preventive measures.^13^

In previous research about GPs' perceptions on the CP's role, GPs described the dispensing of drugs as the main role of CPs.^24^ Additionally, they mentioned that CPs play a role in providing information on drug availability and in conducting medication management reviews. In a case study of CPs' current and potential roles in prescribing in the Netherlands, some GPs seemed open towards a restricted form of prescribing for minor ailments or uncomplicated health conditions, whereas other GPs opposed this idea.^27^ Collaboration between pharmacists and physicians and trust by physicians and patients in pharmacists were deemed essential to support pharmacist prescribing.^27^ In our study, GPs emphasized that CPs could also have an important role in the signaling of long-term antibiotic treatment. This role is an important one, since most GPs prescribe prophylactic treatment to eligible patients with recurrent AC, but sometimes forget to evaluate its need at follow-up.

In some countries CPs already play a role in the diagnosis and treatment initiation of AC.^10^^,^^28^ In those countries, CPs' prescribing authority may contribute to implementation of a pharmacy-led AC service. In the United Kingdom for example, pharmacists can prescribe drugs without supervision of another HCP.^28^ Contrary to this, our results show that implementing a service for AC in community pharmacies seems difficult to realize in the Netherlands at this moment. A major reason for this is the currently experienced lack of time due to staff shortages in the community pharmacies.^25^^,^^29^

### 4.1 Strengths and limitations

A major strength of our study was that data were collected in two ways: through focus groups and individual interviews. The integration of focus groups into the PTAM meeting appeared a successful method to investigate both GPs' and CP's perspectives simultaneously and include a high number of participants. However, a few PTAM groups consisted of a high number of participants, therefore the perspectives of some participants remained in the background. Another limitation of this study was that a mix of GPs and CPs participated in the same focus groups. Because of the presence of collaborating partners, fear of judgement could have led to response bias. Also, the number of GPs attending the focus groups was higher than CPs, and therefore their views were dominant during the focus groups. However, the individual interviews gave the participants the opportunity to freely speak their mind. Also, the individual interviews allowed us to gain insight into the CPs' perspectives if their perspectives did not sufficiently came forward during the focus groups.

Another strength was that we included both GPs and CPs to gain insight into both of their perspectives on the collaboration of AC care and task shifting towards the community pharmacy. The demographic characteristics of participants were representative for GPs and CPs in the Netherlands with regard to gender.^30^ Furthermore, PTAM groups from different areas in the Netherlands were included, amplifying generalizability of the results.

A last strength was that by conducting five focus groups, we were able to reach data saturation in the focus groups. However, due to the limited number of four interviews with CPs, we did not fully reach data saturation with regard to their views.

Members of PTAM groups were approached through the researchers' network and invited to join the study. Recruitment bias could therefore be present, since these HCPs showed their motivation and interest in the research topic. On the other hand, the PTAM groups also consisted of many other members who were also included in the focus groups, and of whom many showed not to be specifically interested in the research topic.

### 4.2 Implications for practice and/or policy

This study provides information to HCPs and policymakers about barriers that should be overcome if task shifting of AC care towards the community pharmacy is considered. Important requirements were: enough pharmacy staff that is able to deliver the service, adequate training of pharmacy staff to ensure they have the knowledge and skills, smooth collaboration and agreements between CPs and GPs, and patients must be informed about the new service, otherwise they will not visit the community pharmacy with their symptoms of AC.

This study also gave insight into other possibilities to improve the collaboration of AC care. HCPs should focus more on the provision of selfcare and preventive recommendations to their patients with AC, and education of support staff might be needed. Additionally, agreements could be made between GPs and CPs about the evaluation of prophylactic antibiotic treatment.

Future research should investigate the perspectives of pharmacy technicians and general practice assistants on task shifting of AC care towards the community pharmacy as well. Also, future research should evaluate the implementation of new services to manage AC in community pharmacies, e.g., as will be performed in France.^31^

## 5 Conclusions

GPs are experienced with providing AC care and delegate most of the tasks to their assistants. There is room for improvement with regard to recommending patients about selfcare and preventive measures, and the minimization of false-negative diagnoses. HCPs saw potential in the use of the ACSS questionnaire for diagnosis, but diagnosis by means of urine testing remained their preference. To make task shifting of AC care towards the community pharmacy possible, some major barriers should be overcome first.

## Declaration of generative AI in scientific writing

During the preparation of this work the authors used ChatGPT in order to improve the readability and make the work more concise. After using this tool/service, the authors reviewed and edited the content as needed and take full responsibility for the content of the publication.

## CRediT authorship contribution statement

**Marle Gemmeke:** Writing – review & editing, Supervision, Methodology, Investigation, Formal analysis, Conceptualization. **Maureen C. Jansen:** Writing – original draft, Investigation, Formal analysis, Data curation. **Thomas G.H. Kempen:** Writing – review & editing, Supervision, Methodology, Conceptualization. **Marcel L. Bouvy:** Writing – review & editing, Methodology, Investigation, Conceptualization.

## Funding

This research received no specific grant from any funding agency in the public, commercial, or not-for-profit sectors.

## Declaration of competing interest

The authors declare that they have no known competing financial interests or personal relationships that could have appeared to influence the work reported in this paper.

## Data Availability

Data available on request due to privacy/ethical restrictions.

## Appendix A Supplementary data

Supplementary material Image 1
