# Supplementary material for: Healthcare providers' perspectives on collaboration of care for acute cystitis in women and the role of the community pharmacy: A qualitative study
Source: Explor Res Clin Soc Pharm. 2025 Dec 6;21:100694. doi: 10.1016/j.rcsop.2025.100694 (PMC12757607; doi:10.1016/j.rcsop.2025.100694)
Supplement: Supplementary file 1 — Supplementary material [file mmc1.docx]

# Appendix A – COREQ checklist

| No | Item | Guide questions/description | Check? |
| --- | --- | --- | --- |
| Domain 1: Research team and reflexivity | | | |
| Personal Characteristics | | | |
| 1. | Interviewer/ facilitator | Which author(s) conducted the interview or focus group? | MJ (PTAMs/interviews)  MG (PTAMs)  MB (PTAMs) |
| 2. | Credentials | What were the researcher’s credentials? | MJ (BSc)   - MSc student - Female   MG (PhD)   - Assistant professor, division of Pharmacoepidemiology and Clinical Pharmacology, UU - Female   MB (PhD)   - Professor pharmaceutical patient care, UU - Male   TK (PhD)   - Assistant professor, division of Pharmacoepidemiology and Clinical Pharmacology, UU & visiting researcher social pharmacy Uppsala University - Male |
| 3. | Occupation | What was their occupation at the time of the study |  |
| 4. | Gender | Was the researcher male or female |  |
| 5. | Experience and training | What experience or training did the researcher have? | Researchers are experienced with qualitative research. The master’s student pharmaceutics had no experience. |
| Relationship with participants | | | |
| 6. | Relationship established | Was a relationship established prior to study commencement? | A few participating pharmacists were familiar to the researchers. |
| 7. | Participant knowledge of the interviewer | What did the participants know about the researcher? | Background and current function. |
| 8. | Interviewer characteristics | What characteristics were reported about the interviewer/facilitator? | Researchers were interested in this study, because they believe that the community pharmacy could play a bigger role in diagnosis and treatment of AC. |
| Domain 2: Study design | | | |
| Theoretical framework | | | |
| 9. | Methodological orientation and theory | What methodological orientation was stated to underpin the study? | The Consolidated Framework for Implementation Research (CFIR) was used. |
| Participant selection | | | |
| 10. | Sampling | How were participants selected? | Participants were recruited through researchers’ network. |
| 11. | Method of approach | How were participants approached? | Based on responses to the advertisement, appointments for the PTAM had been made. Participants were recruited for the interviews afterwards. |
| 12. | Sample size | How many participants were in the study? | In the 5 PTAMs 54 healthcare providers participated, 12 in the interviews. 9 participated in both. Total = 57 participants. |
| 13. | Non-participation | How many people refused to participate or dropped out? Reasons? | None. |
| Setting | | | |
| 14. | Setting of data collection | Where was the data collected? | In the work sphere. |
| 15. | Presence of non-participants | Was anyone else present besides the participants and researchers? | Two researchers were present with the PTAMs. Interviews were done by only one researcher. |
| 16. | Description of sample | What are the important characteristics of the sample? | Gender, years of work experience and position. |
| Data collection | | | |
| 17. | Interview guide | Were questions, prompts, guides provided by the authors? Was it pilot tested? | The interview guide was pilot tested before the interviews. |
| 18. | Repeat interviews | Were repeat interviews carried out? If yes, how many? | No. |
| 19. | Audio/visual recording | Did the research use audio or visual recording to collect the data? | Audio-recording. |
| 20. | Field notes | Were field notes made during and/or after the interview or focus group? | No. |
| 21. | Duration | What was the duration of the interviews or focus group? | PTAM: 1-1,5 hour.  Interview: 20 minutes. |
| 22. | Data saturation | Was data saturation discussed? | Data saturation was not discussed in the study protocol. With five PTAMs, data saturation was met, since no new information came forward. Data saturation was not met with the interviews. These were only explorative, additionally to the PTAMs. |
| 23. | Transcripts returned | Were transcripts returned to participants for comment and/or correction | Participants were aware of their rights to request and check the interview transcript. |
| Domain 3: Analysis and findings | | | |
| Data analysis | | | |
| 24. | Number of data coders | How many data coders coded the data? | Two. |
| 25. | Description of the coding tree | Did authors provide a description of the coding tree? | CFIR was used for the coding tree. |
| 26. | Derivation of themes | Were themes identified in advance or derived from the data? | Deductive coding took place based on CFIR combined with inductive coding. |
| 27. | Software | What software, if applicable, was used to manage the data? | NVivo15. |
| 28. | Participant checking | Did participants provide feedback on the findings? | No. |
| Reporting | | | |
| 29. | Quotations presented | Were participant quotations presented to illustrate the themes/finding? Was each quotation identified? | Participant quotations were used, and each quotation was identified. |
| 30. | Data and findings consistent | Was there consistency between the data presented and the findings? | There was consistency between the data presented and the findings. |
| 31. | Clarity of major themes | Were major themes clearly presented in the findings? | Major themes were clearly presented. |
| 32. | Clarity of minor themes | Is there a description of diverse cases or discussion of minor themes? | Minor themes were mentioned in the appendices. |

# Appendix B – Focus group guide

###### Time used for diagnosis and treatment

1. When does the patient visit to the GP?
2. How long does it take to diagnose AC in your practice? (handing in urine, testing samples, diagnosing)
   1. What is the impact of this process on the patient?

###### Diagnosing

1. What are your experiences with the nitrite test
   1. (Dis)advantages
   2. Are patients asked if they urinated at night?
2. To which extent do you diagnose without an urine test?
   1. Which considerations play a role?
   2. Are you familiar with the criteria in the Dutch GP-guideline?

###### Considerations when deciding whether or not to treat

1. Which treatment options do you consider in your practice?
2. To which extent do you recommend painkillers?
3. To which extent does antibiotic resistance lead to restraints in prescribing antibiotics?
4. Which patient characteristics are important when deciding on treatment? (Severity of infection, history, preference of the patient)

###### Non-pharmacotherapeutic recommendations

1. To what extent are patients familiar with the recommendations?
2. How could this be improved?

###### Prophylaxis

1. Do you ever initiate prophylactic treatment?
   1. Which?
2. Which considerations do you make?
   1. When is the evaluation?

###### Role of the community pharmacy

1. What is the role of the community pharmacy within the care process of AC?
2. Do you think the CP can potentially diagnose someone with a recurrence, based on symptoms?
3. To which extent can a CP inform patients about treatment/selfcare/prevention?
   1. What is necessary to reach this?
   2. What would the added value be?

###### Agreements

1. *Application of wait-and-see policy:* In which cases can you use wait-and-see policy?
2. *Selfcare and lifestyle recommendations:* Which recommendations can be given to the patient and by who?
3. *Application of prophylaxis:* In which cases is prophylaxis applicable?
4. *Application of prophylaxis:* What type of prophylaxis do you apply?
5. *Application of prophylaxis:* When do you evaluate the prophylactic treatment?

# Appendix C – Interview Guide (CFIR)

1. What is your opinion on this new innovative care process? (e.g. difficulties to adjust the current care process, reaching patients, cooperation)
2. What is, according to you, the benefit when the care process for AC would be organized in this way?
   1. What would be the benefit for you/your colleague/the patient? (time, work pleasure, cooperation, etc.)
   2. What could be disadvantages?
3. What do you identify as the main obstacle in the design of this service?
   1. If necessary, how could the service be adjusted to fit implementation?
4. When you look at the current care process for AC in your organization, to which extent is a change of this process necessary?
5. To what extent can this new workflow be carried out within your organization?
   1. What barriers do you expect with implementation?
   2. What consequences does this workflow have on the workload in your own organization?
   3. How well does this new workflow fit with current other workflows in your organization?
6. Which adjustments are needed in your organization to correctly perform this method?
   1. What education is needed for employees to perform this method?
   2. Which additional IT-facilities are necessary in your organization?
   3. Which other resources/space/materials do you need?
7. How is your relationship with the GP/CP you will be working together with, when implementing this method?
   1. Do you expect the GP/CP to be open to this partnership?
8. Which adjustments are needed in the partnership, to perform the new working method well?
   1. Which barriers do you expect with the partnership?
   2. Which agreements need to be made? (Is this possible)
   3. Which IT-connections are needed within the partnership? (and which do you already have)
9. What are possible barriers for patients with this new workflow?
10. How confident are you about this method? (partnership, own role, competences)
    1. Do you expect your colleagues to be willing to participate?
11. How would you make sure the new method is supported/implemented by all your colleagues?
    1. To which extent do you want to contribute and in which way? (e.g. work meetings)
12. How would you make sure the whole partnership supports/implements the new method?
    1. To which extent do you want to contribute and in which way? (e.g. meetings)

| Innovation domain |
| --- |
| What is your opinion on this new innovative care process? |
| What is, according to you, the benefit when the care process for AC would be organized in this way? |
| What do you identify as the main obstacle in the design of this service? |
| **Outer Setting domain** |
| How is your relationship with the GP/CP you will be working together with, when implementing this method? |
| Which adjustments are needed in the partnership, to perform the new working method well? |
| **Inner setting domain** |
| When you look at the current care process for AC in your organization, to which extent is a change necessary? |
| To which extent can this new workflow be carried out within your organization? |
| Which adjustments are needed in your organization to correctly perform this method? |
| **Individuals domain** |
| How confident are you about this method? |
| What are possible barriers for patients, with this new working method? |
| **Implementation process** |
| How would you make sure the new method is supported/implemented by all your colleagues? |
| How would you make sure the whole partnership supports/implements the new method? |

# Appendix D – Schematic design of the innovative care process for AC


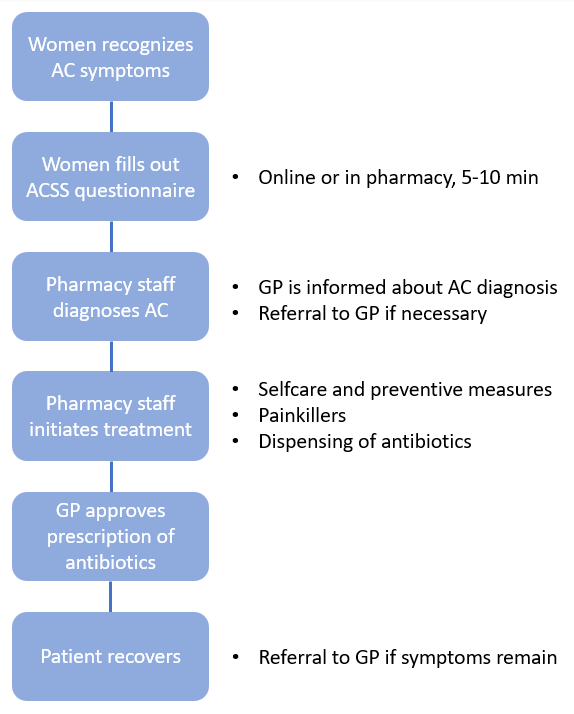


# Appendix E – List of topics

| ***Topic 1:* First contact with the GP** | | | | | | |
| --- | --- | --- | --- | --- | --- | --- |
| Depends on the patient | Patients decide to wait themselves | | | | Variably | |
|  | Women with recurrence visit the GP faster | | | | | |
| Duration of symptoms is unknown | Is asked | Is not asked | | | | |
| Patients often call before visiting the GP | | | | | | |
| ***Topic 2:* Time it takes to diagnose a patient** | | | | | | |
| The same day | Stick + sediment | | | | | |
| The next day or later | | | | | | |
| Time burden diagnosing for GPA | | | | | | |
| ***Topic 3:* Urine testing** | | | | | | |
| Quality of urine | Not always morning urine | | Asking how long urine was in the bladder | | | |
| Negative nitrite | Majority truly negative | | False negative | | | |
|  | Additional testing often needed | | | | | |
| ***Topic 4:* Diagnosis based on symptom recognition** | | | | | | |
| Urine testing is always preferent | | | | | | |
| Risks with diagnosing without urine testing | | | | | | |
| Sometimes diagnosis based on symptoms | | | | | | |
| ***Topic 5:* Choice of treatment** | | | | | | |
| Application of wait-and-see policy | Unfrequently | | Is recommended | | | |
|  | Unclear whether the GPA communicates this | | | | | |
| Application of postponed prescription | Frequency of initiating | | | | | |
|  | Unclear to which extent patients comply to this | | | | | |
| Treatment with antibiotics | With positive diagnosis almost always antibiotics | | | | | |
|  | Self-initiated treatment | | | | | |
|  | Most antibiotics are collected from the community pharmacy | | | | | |
| Considerations when deciding on treatment | | | | | | |
| ***Topic 6:* Selfcare and lifestyle recommendations** | | | | | | |
| Talk about recommendations with recurrence | | | | | | |
| Materials | Thuisarts.nl | | | Other | | |
| Patient not always familiar with recommendations | Information does not reach the patient | | | | | |
| Painkillers | Frequency of recommending painkillers | | | | | |
|  | Unclear to which extent this is recommended | | | | | |
| Role for the GPA | Check how GPAs handle giving recommendations | | | | | |
|  | Knowledge of the GPA | | | Possible contribution | | |
| Self-care products | Cranberry | | | Estradiol | | |
|  | D-mannose | | | Other | | |
| ***Topic 7:* Prophylaxis** | | | | | | |
| Prophylaxis is applied (frequently) | Continuous prophylaxis | | | Postcoital prophylaxis | | |
| Considerations with prophylaxis | Giving lifestyle recommendations before initiating prophylaxis | | | | | Risks |
| Evaluation of prophylaxis | Usage longer than 6-12 months takes place | | | | | |
|  | Easy to evaluate | | | Evaluation is standard | | |
| ***Topic 8:* Role of the community pharmacy** | | | | | | |
| Providing medication | | | | | | |
| Recommendations from the community pharmacy | Recommendations with first dispense | | | What is not discussed | | |
|  | Twice is unnecessary | | | Meaningful with first dispense | | |
| Signaling function | Prophylaxis is used long-term, evaluation needed | | | | | |
| Task shifting towards community pharmacy | Community pharmacy is not suitable | | | | | |
|  | Additional value of task shifting still unclear | | | | | |
|  | Disadvantages of task shifting | | | Requirements for task shifting | | |
